# Detecting shapes in noise: tuning characteristics of global shape mechanisms

**DOI:** 10.3389/fncom.2013.00037

**Published:** 2013-05-16

**Authors:** Gunnar Schmidtmann, Gael E. Gordon, David M. Bennett, Gunter Loffler

**Affiliations:** Vision Sciences, Glasgow Caledonian UniversityGlasgow, UK

**Keywords:** form, contour, global shape, signal integration, texture

## Abstract

The proportion of signal elements embedded in noise needed to detect a signal is a standard tool for investigating motion perception. This paradigm was applied to the shape domain to determine how local information is pooled into a global percept. Stimulus arrays consisted of oriented Gabor elements that sampled the circumference of concentric radial frequency (RF) patterns. Individual Gabors were oriented tangentially to the shape (signal) or randomly (noise). In different conditions, signal elements were located randomly within the entire array or constrained to fall along one of the concentric contours. Coherence thresholds were measured for RF patterns with various frequencies (number of corners) and amplitudes (“sharpness” of corners). Coherence thresholds (about 10% = 15 elements) were lowest for circular shapes. Manipulating shape frequency or amplitude showed a range where thresholds remain unaffected (frequency ≤ RF4; amplitude ≤ 0.05). Increasing either parameter caused thresholds to rise. Compared to circles, thresholds increased by approximately four times for RF13 and five times for amplitudes of 0.3. Confining the signals to individual contours significantly reduced the number of elements needed to reach threshold (between 4 and 6), independent of the total number of elements on the contour or contour shape. Finally, adding external noise to the orientation of the elements had a greater effect on detection thresholds than adding noise to their position. These results provide evidence for a series of highly sensitive, shape-specific analysers which sum information globally but only from within specific annuli. These global mechanisms are tuned to position and orientation of local elements from which they pool information. The overall performance for arrays of elements can be explained by the sensitivity of multiple, independent concentric shape detectors rather than a single detector integrating information widely across space (e.g. Glass pattern detector).

## Introduction

It is well-established that neurons at the early stages of cortical visual processing only respond to small fractions of the visual scene. At the first cortical level (V1), these neurons act like filters tuned to contour orientation and scale (Hubel and Wiesel, 1962). At subsequent stages along the ventral processing stream (Ungerleider and Mishkin, 1982; Goodale and Milner, 1992) neurons become selective for more complex features including contour curvature, angles, and contour arcs (Dobbins et al., 1987; Hegde and Van Essen, 2000; Pasupathy and Connor, 2001). At the highest stages within the ventral stream [inferotemporal cortex (IT) and lateral occipital complex (LOC)], neurons have been shown to be selective for extended and highly complex stimuli including faces and objects (Goodale and Milner, 1992; Tanaka, 1996). If cells at the early stages of the visual system are selective for simple features like contour orientation, the question arises as to how this local information is integrated to represent more complex object properties at subsequent stages.

Field et al. (1993) proposed a paradigm that has been used widely to investigate a first stage of this integration process. This paradigm required observers to detect a contour path of aligned Gabors in an array of randomly orientated Gabors. Field et al. (1993) demonstrated that detection is strongly dependent on the relative orientation between adjacent elements that make up the path. If the orientation of neighboring elements exceeds about 30°, the path becomes invisible. Lateral connections between cells with similar orientation preferences in V1 have been suggested as a plausible neuronal substrate for these results (Li and Gilbert, 2002).

Beyond the connections between neurons with adjunct receptive fields in V1, studies have looked at how the visual system integrates information over wider distances to represent more extended patterns. One popular approach has been to determine the minimum number of signal elements required to detect the presence of a global pattern embedded in arrays of noise elements. Determining coherence thresholds has been used successfully in a range of studies on motion (Newsome and Pare, 1988; Braddick et al., 2000), texture (Dakin, 1997; Wilson et al., 1997; Wilson and Wilkinson, 1998) and form perception (Braddick et al., 2000; Achtman et al., 2003). One aim of the current study was to investigate the strategy used by the visual system to detect form structure in noise. A potential strategy involves the use of texture detectors. Glass patterns (Glass, 1969) have been used frequently to investigate signal integration in texture perception (Dakin, 1997; Wilson et al., 1997; Wilson and Wilkinson, 1998; Dakin and Bex, 2002). Glass patterns are composed of an array of randomly positioned dot pairs. The spatial relationship (orientation given by the invisible line connecting dot pairs) can be used to define the geometry of the global texture. If dot pairs falls on horizontal lines, the resulting pattern contains parallel (translational) horizontal texture. If the orientation of the dot pair is tangential to concentric circles, rotational texture is perceived. It has been shown that sensitivity is highest for concentric/rotational Glass patterns, followed by radial and hyperbolic arrangements and lowest for parallel texture (Wilson et al., 1997; Wilson and Wilkinson, 1998; cf. Dakin and Bex, 2002). Given the arrangement of the dots, the overall geometry of the textures cannot be detected on a purely local level. The visual system must firstly group dots and compute local orientations and then combine the local information across space to yield the global structure. The extent over which signals are pooled across space has been shown to depend on the type of texture. Concentric and radial Glass patterns are integrated across extended regions, more widely than parallel patterns (Wilson et al., 1997).

**Figure 1:**
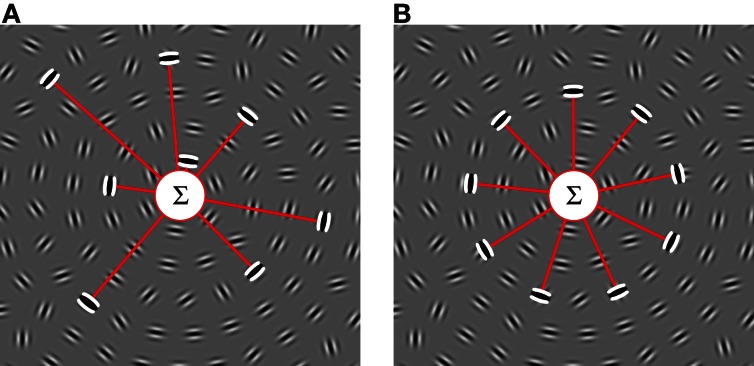
**Schematic models of global signal integration for concentric circular structures in noise.** The gray images show an example of the stimuli used in this study. **(A)** Glass pattern detector (Glass, 1969) selective for global texture. Local contour orientations are processed across the visual field by a bank of V1 cells (illustrated by the triplet of white and black ellipses, which represent on and off regions) and their outputs are summed (Σ) by the global detector (the Glass pattern detector shown here is selective for concentric/rotational texture). Provided that their preferred orientations are perpendicular to radii emerging from the pattern center, the global texture detector integrates information independent of the local position of each individual filter. **(B)** Global shape detector (Wilkinson et al., 1998). Information from local orientation detectors is pooled by a global process as long as their preferred orientations are tangential to the shape (circles in this example) and their positions fall on the circumference of the contour. Both global detectors constrain local orientation preference but only the shape detector poses additional constraints on the location of the local signals. Note that for clarity, the diagrams omit details including rectification and computation of local curvature (Wilkinson et al., 1998; Poirier and Wilson, 2006).

In a modification to the classical Glass pattern, dot pairs were replaced by oriented Gabors (Braddick et al., 2000; Achtman et al., 2003); this avoids the need for local orientations to be computed by grouping dots. In this paradigm, about 10% of signal elements are required to detect circular concentric patterns, however, coherence thresholds rise for radial and spiral configurations (Achtman et al., 2003). Introducing orientation jitter increases coherence thresholds but thresholds are impervious to manipulations of the number, contrast, spatial frequency, polarity, or position of elements. Given the insensitivity to the local position of the elements, Achtman et al. (2003) proposed that the underlying mechanism pooled signals from anywhere within its large receptive field, similar to the mechanism proposed for concentric Glass patterns (Figure 1A; Wilson and Wilkinson, 1998).

An alternative strategy for the detection of form structure in noise is the recruitment of shape processes. In contrast to texture, global shape mechanisms are sensitive to signal position (Keeble and Hess, 1999; Levi and Klein, 2000). Radial frequency (RF) patterns are a class of stimuli that have been used widely to study global shape processing (Wilkinson et al., 1998; Loffler, 2008). RF patterns can be used to represent a variety of natural shapes, including fruits and face contours (Wilson and Wilkinson, 2002; Wilson et al., 2002; Loffler et al., 2003). A number of studies have provided compelling evidence that RF patterns are processed globally by integrating information from the entire contour (Wilkinson et al., 1998; Hess et al., 1999; Loffler et al., 2003; Bell et al., 2007; Dickinson et al., 2012; Schmidtmann et al., 2012). Imaging (fMRI) and physiological studies have implicated extra-striate area V4 in the processing of these types of shapes (Wilkinson et al., 2000; Pasupathy and Connor, 2001).

Models for RF shape processing propose that information is integrated across the circumference of a circular contour as long as the local orientations are tangential to the shape (Figure 1B; Wilkinson et al., 1998; Poirier and Wilson, 2006). These RF shape models differ in a fundamental way from Glass pattern detectors (Figure 1A). Whereas the RF shape detector constrains both element orientation and position, the Glass pattern detector requires element orientations to be concentric but is insensitive to position. The first aim of the current study was to investigate the nature of the mechanism that limits performance when concentric contours are embedded in a background of randomly oriented elements and to distinguish between these two strategies.

The second aim was to determine how the process of signal integration depends on the global shape of the sampled contours. To study a range of shapes we employed circular contours as well as RF patterns with varying number of lobes (e.g., circular or pentagon shapes with 5 lobes) and sharpness (e.g., pentagon with rounded corners or five-sided star shapes).

## Materials and methods

### Observers

Four experienced observers participated in the experiments; one was naïve as to the purpose of the experiments. All observers had normal or corrected-to-normal visual acuity. Experiments were carried out under binocular viewing conditions. No feedback was given either during practice or during the experiments.

### Apparatus

Stimuli were generated within the MatLab environment and presented on a LaCie “electron22blueII” monitor (mean luminance of 65 cd/m^2^) with a spatial and temporal resolution of 1024 × 768 pixels and 85 Hz, respectively. The monitor was gamma-corrected by defining the color lookup in a way that minimizes luminance non-linearities employing routines from the Videotoolbox (Pelli, 1997). A chin and forehead-rest was used to maintain a constant viewing distance of 120 cm. At this distance, one pixel subtended 0.0177°. A circular cardboard mask with a diameter of 12° was positioned in front of the monitor to minimize reference cues. All experiments were carried out under dim background illumination.

### Stimuli

**Figure 2:**
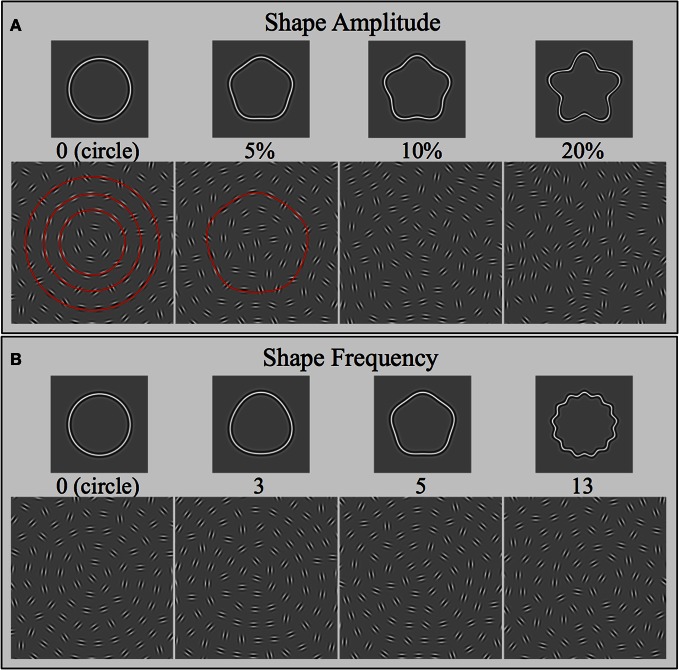
**Stimulus arrays used in this study.** Stimuli consisted of arrays of Gabors placed on a polar grid. Gabors sampled various concentric shapes and were positioned on their circumference. The continuous contours show examples of the sampled radial frequency shapes, varying either in the number of lobes (**B**: Shape Frequency) or the pointedness of each lobe (**A**: Shape Amplitude). The Gabor arrays directly underneath the shapes show the corresponding stimuli presented to the observers. The arrays nominally consisted of multiple concentric shapes. The red contours superimposed on two of the arrays illustrate the concentric nature of the stimuli. Gabor orientation was either tangential to the shapes (signal) or random (noise). In all cases shown here, the coherence level is 50%, i.e., half the elements are oriented tangential to the shapes. Note that it becomes increasingly difficult to perceive the signal with increasing shape frequency and amplitude. The arrays are not to scale and contain fewer elements than in the actual experiments. See text for further details.

Stimuli were presented within square windows (8.9° × 8.9°) and consisted of multiple, concentric shapes, which were sampled by oriented Gabors (Figure 2). The shapes (RF patterns; Wilkinson et al., 1998) were defined by sinusoidal modulations of a radius in polar coordinates:

(1) $$r(\theta )=r_{mean}\cdot (1+A\cdot \sin(\omega \theta +\varphi ))$$

where *r*_*mean*_ represents the mean radius (size), ϕ the phase (orientation), ω the frequency (number of cycles or corners), and *A* the modulation amplitude (pointedness of each corner). Icons at the top of Figure 2B illustrate a range of RF frequencies (e.g., ω = 0 = circle; ω = 5 = pentagon shape; ω = 13 = 13-lobed star); icons at the top of Figure 2A illustrate a range of RF amplitudes for the case of an RF5 pattern (e.g., *A* = 0 = circle; *A* = 5% = pentagon shape without concavities; *A* = 20% = five-sided star).

Shapes were sampled by Gabor elements, where each element is given by:

(2) $$G=c\cdot e^{-\frac{\left(x^{2}\;+\;y^{2}\right)}{2\sigma^{2}}}\cdot \cos(2\pi f(x\cos\phi +y\sin\phi )+\delta )$$

The position of a Gabor is given by (*x, y*) and its orientation by ϕ. Its phase, δ, was set to 0 (cosine phase) and the peak spatial frequency, *f*, set to 6 c/°. The circular-symmetric Gaussian envelope of the Gabors had a standard deviation of σ = 0.1°. The contrast (*c*) was 98%.

The Gabor elements in each stimulus array were positioned according to a polar coordinate system. The radial dimension was given by the circumference of multiple, concentric RF shapes (three concentric circles are shown in red in the left-hand icon in Figure 2A) and the angular dimension by a regularly spaced radial grid. To achieve an approximately equal inter-element spacing throughout the array, the number of Gabors per RF contour and their radii (*r*_*mean*_) were co-varied. For all stimulus arrays, irrespective of the shape of the sampled contours, the number of elements on each ring was fixed. The innermost shape had a radius of 0.72° and was sampled by 6 elements. The radius increased by 0.72° for each successive concentric shape; the number of samples increased by 6. This resulted in 6, 12, 18, 24, 30, and 36 elements for the innermost 6 concentric shapes (rings), all of which were visible within the square array (they are not all visible in Figure 2). This arrangement resulted in an average inter-element spacing between a Gabor and its four closest neighbors of 0.72°. The total number of elements contained within the square window depended on the shape of the concentric contours and ranged from 140 to 160. The concentric contours for any stimulus array were always matched in shape (i.e., same RF frequency, amplitude, and phase). Gabors were spaced equally along each shape but angular positions across concentric shapes were randomized to avoid any obvious regularity within the arrays. All elements were calculated individually for each presentation. Consequently, the position of elements varied randomly between trials. The arrays were presented at the center of the screen with a small, random positional jitter of ±10 pixels (0.177°).

Element position was hence determined by the shape to be tested. Orientation determined whether a Gabor element was signal or noise. Signal elements were oriented tangential to the RF shape, at the point where they were centered. The orientation of noise elements was allocated at random between 1 and 360°. In experiment 1, signal and noise elements were assigned randomly within the entire array. In experiment 2, signal elements were constrained to fall on one of the concentric shapes, however, their position within that shape was selected randomly. This selection results in an interruption of collinear contour elements for all but the 100% coherence levels. For instance, for a contour sampled by 24 elements (ring 4), for a coherence level of 20%, there were, on average, 4 randomly oriented noise elements between any two signal elements.

### Procedure

Using a 2AFC paradigm with a method of constant stimuli, observers were asked to detect which of two successively presented stimulus arrays contained concentric contours. One of the stimuli contained a variable fraction of signal elements, the other contained noise only. Note that the positions of the Gabors within the signal-carrying and noise-only stimuli did not differ apart from the randomizations described above. Hence, observers could not use element position as a cue to the task. Subjects indicated their choice by pressing a key on a computer keyboard. Subsequent trials were initiated by pressing another key. Presentation time was 400 ms. In each experimental block, six levels of coherence (ratio between number of signal elements and the total number of elements) were randomly presented. Each coherence level was presented 30 times, in random order, for a total of 180 trials per condition. Percent correct responses were calculated and the resulting data fit by a Quick function using a maximum-likelihood procedure (Quick, 1974). Coherence detection thresholds were defined as the point on the function at which observers performed at the 75% correct level. The coherence levels selected were appropriate for each condition and each observer. The progression between coherence levels was set to 2 dB. Observers completed short practice runs prior to data collection and repeated each condition at least twice, on separate occasions. Thresholds were averaged across runs. Different shape conditions were run in separate blocks.

## Results

### Experiment 1—dependence of detection thresholds on shape

**Figure 3:**
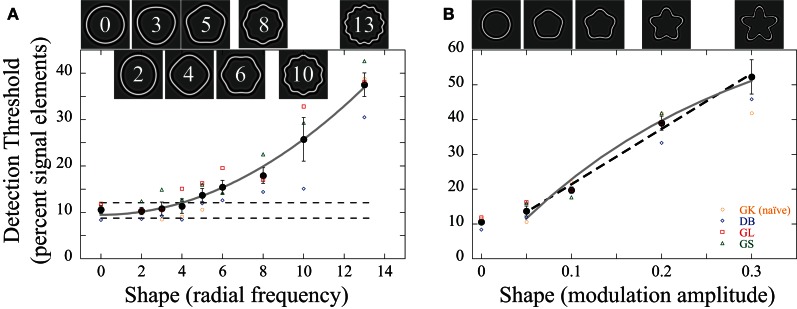
**(A)** Dependence of detection thresholds on shape frequency. The graphs show the individual (colored data points) and average (black filled circles) detection thresholds (the percentage of signal elements relative to all elements in the array) as a function of **(A)** radial frequency and **(B)** amplitude. Four subjects participated; observer GK (orange circles) was naïve as to the purpose of the experiments. The icons above the data illustrate the general shape of the concentric contours that were sampled by Gabors and embedded in noise. **(A)** Shape frequency: the amplitude of the shapes was fixed at *A* = 0.05. Detection thresholds for low RF patterns were ~10% and remained largely unaffected up to RF4 but rose for higher RF patterns (the dashed lines represent the 95% confidence intervals for the circular contours; RF0). Thresholds increased approximately with the square of the shape frequency (solid gray line). **(B)** Shape amplitude (“sharpness” of the lobes) had a negligible effect on performance for low amplitudes but decreased approximately linearly (dashed line) when amplitudes were increased beyond about *A* = 0.05. The solid gray line shows the predicted dependence of thresholds on amplitude based on the curvature maximum of the contour (see Discussion). The error bars here and elsewhere represent the standard error of the mean across observers. As is evident from the graphs, there is no quantitative or qualitative difference between the naïve and the informed observers.

Coherence thresholds were tested for arrays consisting of different concentric RF shapes. In the first condition, the RF frequency (ω, number of corners/lobes) was manipulated. Observers were presented with either an RF0 (circular shape), RF2, RF3, RF4, RF5, RF6, RF8, RF10, or RF13. In order to test non-circular contours the amplitude of all patterns, apart from the circle, was set to *A* = 0.05. This corresponds to approximately 10–15 times the threshold required for normal observers to discriminate single, continuous RF shapes from a circle (Wilkinson et al., 1998; Loffler et al., 2003). Thus, the contours were clearly visible as non-circular shapes. Icons at the top of Figure 3 show the appearance of equivalent closed contour RF shapes.

Figure 3A shows average detection thresholds as well as individual data for four observers defined as the percentage of signal elements required to achieve 75% correct performance as a function of shape frequency. The coherence threshold for circular contours (RF = 0) was 10.6%. This is in line with previous reports (Achtman et al., 2003). About 10% of all Gabors (~15 elements here) need to be oriented tangential to the concentric circles for observers to detect the interval with the signal. This level of performance was independent of shape for frequencies of up to RF4 but thresholds increased for higher radial frequencies. A repeated measures ANOVA with shape frequency as factor showed a main effect [*F*_(8, 24)_ = 43.74; *p* < 0.001]. Throughout the document, we conducted Fisher's least significant difference tests (LSD) to assess differences between individual conditions. According to this, coherence thresholds for RF ≥ 5 are significantly elevated compared to the data for a circle (*p* < 0.05). For the highest RF pattern tested (13), thresholds were almost four times those for circles (38% = 57 signal elements). Coherence thresholds increased approximately with the square of the shape frequency (*R*^2^ = 0.99).

In a second condition, we measured the dependence of detection thresholds on the shape modulation amplitude (*A*) for one frequency (RF5): *A* = 0 (circle), 0.05, 0.1, 0.2, 0.3. There was a negligible difference between the circular shapes (*A* = 0) and the RF5 pattern with an amplitude of *A* = 0.05 (Figure 3B). A repeated measures ANOVA with the modulation amplitude as factor showed a main effect [*F*_(4, 12)_ = 58.684, *p* < 0.001], but *post-hoc* tests revealed no significant difference between circle and *A* = 0.05 (*p* = 0.112). Detection thresholds above *A* = 0.05 increased approximately linearly with increasing modulation amplitude (*R*^2^ = 0.99; dashed line in Figure 3B). Taken together, there appears to be a range where shape has little effect on detection thresholds. This is the case for shape amplitude (*A* = 0.05) as well as frequency (ω ≤ 4). Increasing the number or sharpness of lobes beyond this decreases sensitivity.

### Experiment 2—strategy of signal integration

The aim of the second experiment was to distinguish between two plausible mechanisms underlying the signal integration in this task (see Figure 1). The first candidate is a mechanism that is tuned for contour shape and requires local information to be specific with regards to the position and orientation of local signals that feed into it (Figure 1B). The second candidate mechanism is a texture detector (Glass pattern detector), which is tuned to orientation but insensitive to local position (Figure 1A).

To distinguish between global texture and global shape mechanisms, we manipulated the location of signal elements, either spreading them randomly across the stimulus region or constraining them to fall along individual concentric rings. A global texture detector should be insensitive to the location of signal elements within its receptive field and hence the performance for the two manipulations should be the same. On the other hand, a global shape detector that only sums information along the contour for which it is tuned would require fewer signal elements if they fell on the contour than if they were spread randomly.

**Figure 4:**
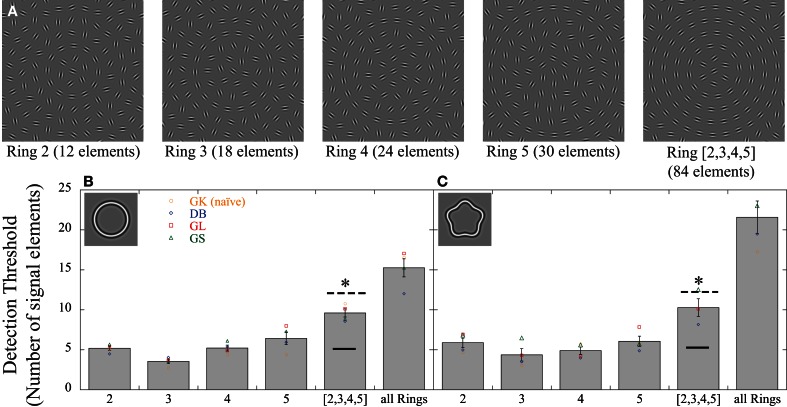
**Effect of signal element distribution. (A)** Examples of stimuli used to investigate strategies of signal integration. Performance was compared between conditions where signals were randomly positioned across rings ([2,3,4,5]) to conditions where signals were constrained to fall on individual rings. Individual rings contain different number of elements (12 for ring 2; 18 for ring 3; 24 for ring 4; and 30 for ring 5). The graphs **(B,C)** show the individual (colored data points) and average (gray bars) detection thresholds (number of signal elements). Four subjects were tested; GK (orange circles) was naïve. **(B)** For circular contours, few signal elements were required when they fell on individual rings (between 3.56 and 6.44). Significantly more elements were needed to reach threshold when signal elements were randomly spread across the entire array (“all Rings”) or across the four rings ([2,3,4,5]). The prediction of probability summation over multiple, independent concentric detectors, each integrating information along individual contours (dashed line) correctly predicts increased thresholds for the spread condition but slightly underestimates actual sensitivity. The prediction of a perfect linear integrator is indicated by the solid horizontal line. It is obvious that the data for the spread condition are inconsistent with this prediction. **(C)** The same pattern was seen for a different shape (RF5; *A* = 0.05). The asterisks indicate significant differences (*p* < 0.05) between any of the four individual rings and the data where elements were spread.

In experiment 1, signal elements were randomly allocated to any of the Gabors in the array. In the experiment here, signal elements were constrained to individual contours (i.e., one of the concentric shapes). Sensitivity was measured for four radii (rings) (Figure 4): ring 2 (second innermost of the sampled concentric contours), which contained 12 elements, ring 3 (18 elements), ring 4 (24 elements), and ring 5 (30 elements). Signal elements were randomly selected from the respective ring elements. Performance was also measured when the signal elements were randomly selected from any of these four rings (84 elements). Data are reported as the number of signal elements to reach threshold rather than the coherence levels as in experiment 1, to simplify the comparison between rings. Performance for the four rings was measured in a single block as four, randomly interleaved conditions. Thus, observers were unable to predict where signal elements would occur on a trial-to-trial basis. Thresholds were measured separately for two sets of concentric shapes: a circle and an RF5 pattern with *A* = 0.05.

For the circle, neither the size of the contour, nor the total number of elements on it, correlated monotonically with the number of signal elements needed to detect the target ring (Figure 4B). The number of signal elements at threshold was 5.20 ± 0.49 (95% confidence intervals; CI) for the contour with 12 elements and a radius of 1.44° and 6.44 ± 1.56 for the contour with 30 elements and a radius of 3.60°. The fewest elements were required for an intermediate ring (3.56 ± 0.54 for ring 3 with 18 elements and a radius of 2.16°). A repeated measures ANOVA with contour rings as factor showed a significant effect [*F*_(4, 12)_ = 22.43, *p* < 0.001]. *Post-hoc* tests revealed no significant differences between any of the rings with the exception of ring 3, which required fewer elements than the other rings (*p* < 0.05).

More importantly, compared to each individual ring, significantly more elements are needed when signals were randomly distributed across the entire array (15.25 ± 2.21; “all Rings” in Figure 4B) or across the four rings (9.62 ± 1.02; “[2,3,4,5]”). This argues against a mechanism that integrates information linearly across the entire display (texture detector) and instead suggests that information is integrated most efficiently when signals fall on individual contours. We calculated the number of predicted signal elements for the condition where the signal elements are spread randomly, based on the actual performance for the individual rings. The calculation assumed that four independent detectors simultaneously process the entire display, each summing information within an annular region, i.e., along a contour. If a global process could sum the outputs from such detectors linearly prior to their individual thresholds, the same signal elements would be required to reach threshold, regardless of how they are distributed. This mechanism makes the same prediction as the texture detector (Figure 1A; black horizontal line), inconsistent with the data. An alternative prediction can be based on the assumption that individual contour detectors are subject to independent noise sources, and the overall performance is given by probability summation of their outputs (Graham and Robson, 1987; Morrone et al., 1995; Loffler et al., 2003). We calculated the prediction of probability summation, assuming 4 independent contour detectors, each limited by its own noise, in the following way:

(3) $$S_{\text{ProbSum}}={\left[\sum_{i\;=\;1}^{N}{\left(S_{i}\right)}^{\beta }\right]}^{\frac{1}{\beta }}$$

*S*_ProbSum_ is the predicted sensitivity when elements are spread across the array, *S*_*i*_ is the sensitivity of the *i*-th contour detector (calculated as the inverse of the signal-to-noise ratio at threshold), *N* is the number of detectors (4 in this case) and β is the average slope of the psychometric function. For the current experiments β was close to 3, in agreement with similar studies (Morrone et al., 1995; Loffler et al., 2003). This equation is a general form of:

(3a) $$S_{\text{Pred}}=k\cdot Ar^{1/\beta }$$

where *k* is an arbitrary constant and *Ar* corresponds to stimulus area. This equation has been widely used to describe the modest improvement in sensitivity with increasing stimulus area (Graham and Robson, 1987; Morrone et al., 1995; Loffler et al., 2003), under the assumption of equal sensitivity of local detectors. We applied Equation 3 to the data given that the sensitivity for individual contour rings cannot be assumed equal in our case. The prediction, shown by the dashed line in Figure 4B, is close to but slightly underestimates performance when signals are spread across rings (“[2,3,4,5]”). However, probability summation is a sub-ideal strategy to combine information from multiple, independent sources (Macmillan and Creelman, 1991). Other strategies (e.g., integration rule—Macmillan and Creelman, 1991; information summation—Machilsen and Wagemans, 2011; optimal Bayesian integrator—Nandy and Tjan, 2008; Gold et al., 2012) predict slightly better performance in a compound condition. Analysis (e.g., for an optimal Bayesian integrator by setting β = 2 in Equation 3; Nandy and Tjan, 2008; Gold et al., 2012) shows that this can account for the performance in the spread condition. Irrespective of the strategy used, it is clear that a single, global process that linearly sums information across the entire display is inconsistent with our data. Instead, the performance when signals are spread across rings (“[2,3,4,5]”) argues in favor of multiple, independent contour detectors. Each of these detectors integrates information only within an annular region.

This pattern of results was independent of contour shape: repeating the experiment with concentric RF5 contours yields very similar data (Figure 4C). The data for individual rings ranged between 4.37 ± 1.48 (CI) and 6.08 ± 1.24 signal elements compared to 10.08 ± 1.79 when signals are spread across rings (“[2,3,4,5]”). Statistical analysis [*F*_(4, 12)_ = 18.12, *p* < 0.001] showed a significant difference (*p* < 0.05) between each individual ring and the spread condition. As for the circular shape, the prediction of probability summation over independent contour detectors (in this case with shapes given by an RF5) is close, but slightly underestimates the performance for the condition where elements are spread across the array. This suggests that the visual system may engage a more efficient strategy than probability summation to combine the information from individual rings. Irrespective of the details of the strategy, the significantly higher thresholds for the condition when elements are spread across rings compared to when they are constrained to individual rings show that signal integration across rings is different and gives poorer performance than signal integration within rings.

### Experiment 3—tuning characteristics

#### Effect of orientation jitter

**Figure 5:**
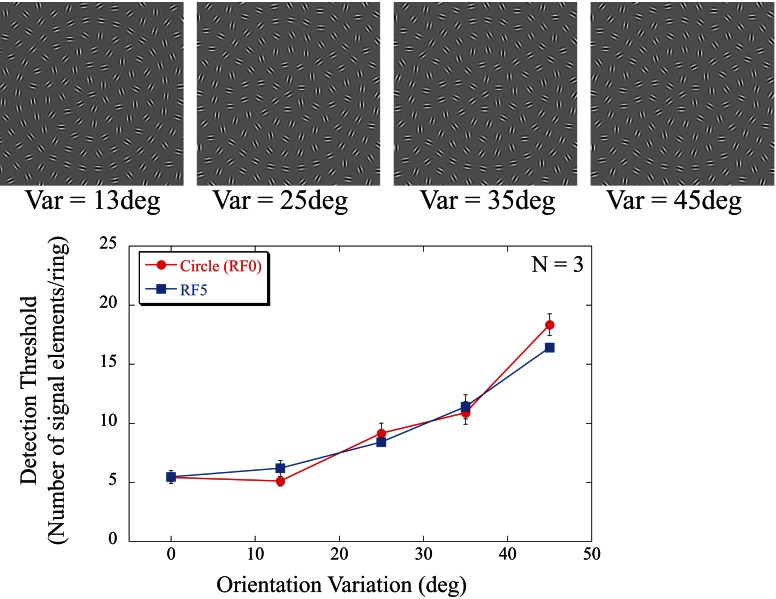
**Dependence of detection of circles (red line and circular symbols) and RF5 shapes (blue line and squares) on random orientation variation added to the signal elements.** The icons at the top show examples of contours with different amounts of orientation variance. The examples are for circular shapes and for ring 5 (fifth contour from the stimulus center). All 30 elements of that ring are signal elements in these icons with their orientations drawn from a uniform distribution centered at the tangential orientation with different amounts of variance. With sufficient orientation variance (Var = ±45°), the circular contour is difficult to see even if all elements are nominally signal. The data (averaged across observers and rings) show that a small orientation variance of ±13° did not affect detection thresholds but performance deteriorated with higher variances (±25, ±35, and ±45°). This was independent of shape. Data for a circle and the RF5 pattern were essentially indistinguishable.

The aim of the final experiment was to investigate the tuning characteristics of the mechanisms responsible for sampling local information along contours by adding external noise to the signal elements. In the first part, noise was added to the orientation of the signal elements. Detection thresholds were measured in the presence of a series of orientation jitter levels for two shapes (circles and RF5 with *A* = 0.05). Stimulus arrays were created in the same way as in experiment 2, with signal elements selected randomly from individual rings and thresholds determined separately for each of four contour rings (ring 2 to ring 5), randomly interleaved in a single block. This time, instead of signal elements being oriented tangentially to the contour (experiment 1 and 2), here orientations were selected from a uniform distribution with a mean of 0 (tangential to the contour shape) and spreads of 0° (no added orientation jitter), ±13, ±25, or ± 45° (Figure 5). As in experiment 2, sensitivity was defined as the number of signal elements required to detect the signal array.

Detection thresholds show little dependence on orientation jitter for variances of ±13°. Differences in performance were analysed with a repeated measures ANOVA, with shapes (circle, RF5), rings (2, 3, 4, 5) and orientation jitter (0°, 13°, 25°, 35°, 45°) as factors. This showed no significant difference between the two shapes [*F*_(1, 24)_ = 2.170, *p* = 0.279] or the individual rings [*F*_(3, 24)_ = 2.741, *p* = 0.136], but a significant main effect for orientation jitter [*F*_(4, 24)_ = 318.1, *p* < 0.001]. *Post-hoc* tests revealed no significant differences between orientation jitters of 0° and ±13° (*p* = 0.358), however, performance significantly decreases for higher jitters (*p* < 0.05).

This suggests that the underlying mechanisms are insensitive to moderate amounts of orientation noise and that elements with orientations within ±13° of being tangential to the contour are globally integrated.

#### Effect of positional jitter

**Figure 6:**
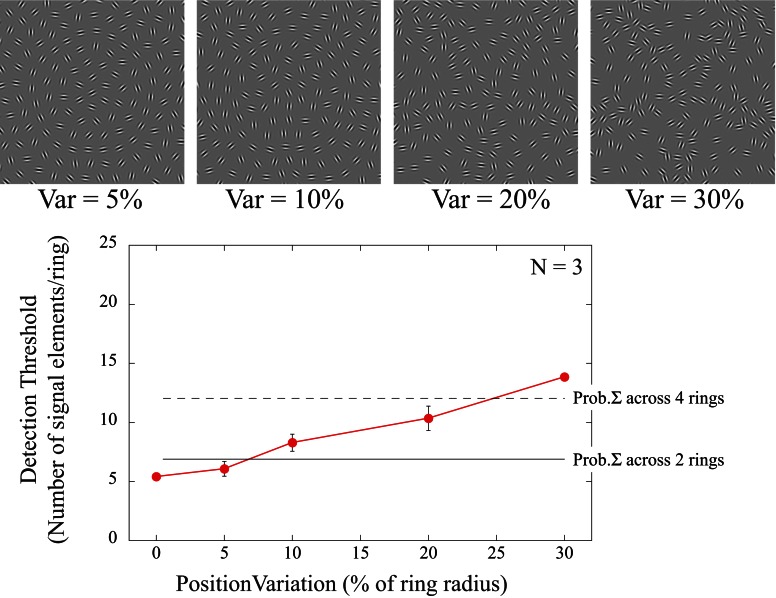
**Dependence of detection threshold on radial noise added to the position of the elements.** The icons at the top show examples of different amounts of positional variance added to a circular shape (ring 4). All 24 elements of that ring are signal elements in these icons. The signal orientations are tangential to the circular contour but their positions are sampled from RF5 shapes with varying amplitudes (*A* = 0.05; 0.1, 0.2, and 0.3) corresponding to radial variations of 5, 10, 20, and 30% of the contour radius. For a variance of 0°, signal elements are positioned on a circle with orientations sampled from a circle (data re-plotted from Figure 3A). Positional jitter of 5% did not significantly increase detection thresholds but larger positional variance reduced detectability. The thin horizontal lines are the predictions for the case of multiple, independent, concentric shape detectors that encode the stimulus array simultaneously. Based on the threshold for the baseline condition (0% positional variance), if elements were spread across a number of independent detectors, the total number of elements required to reach thresholds can be calculated by probability summation. The two lines illustrate the predictions for 2 (solid) and 4 (dashed) independent detectors respectively.

In the second part of experiment 3, positional noise was added to the signal elements. Detection thresholds were determined for signal elements with orientations tangential to a circle but with radial jitter added to their positions. To retain a regular array and avoid element overlap with increasing amounts of radial noise, the position of all array elements were selected to fall on circumferences of RF5 shapes with varying amplitude (0.05, 0.1, 0.2, and 0.3) corresponding to radial variations of 5, 10, 20, and 30% of the radius of each concentric circle (Figure 6). As before, thresholds for individual rings were tested separately but interleaved within experimental blocks.

Detection thresholds showed a significant dependence on position noise [*F*_(4, 8)_ = 47.74; *p* < 0.001]. Subjects required on average 5 signal elements to detect the shape when element positions were sampled from a smooth circle (0% position variance). *Post-hoc* tests revealed no significant differences up to 5% positional jitter (*p* = 0.271), suggesting that the underlying mechanism is insensitive to this amount of positional variance. Even for the highest positional noise tested (30%) only around 14 signal elements were needed to detect the target despite the fact that this completely eliminated the contour path of a circle.

The number of signal elements required for detection in the presence of positional noise may be predicted by probability summation. Assuming that multiple, concentric, shape detectors simultaneously encode the stimulus arrays, each independent and limited by its own noise, the two thin lines in Figure 6 illustrate the predictions for 2 (solid) and 4 (dashed) detectors, respectively. Therefore, when elements are spread radially and are considered to fall into the receptive fields of adjacent concentric detectors tuned to different sizes, this prediction provides a reasonable explanation for the subtle increase in detection thresholds with increasing amounts of positional jitter.

## Discussion

### Dependence of shape detection on contour shape

In the first part of this study, we addressed the question of how the detection of Gabor-sampled concentric contours depends on the shape of the contours. In line with earlier results (Achtman et al., 2003), an average of about 10% of signal elements was required to detect circular shapes. These coherence thresholds were somewhat lower than those reported for rotational Glass patterns, which fall in the range of around 15% (Wilson and Wilkinson, 1998; Dakin and Bex, 2002). Substituting the circle for various RF shapes had a systematic effect on coherence thresholds. Whether manipulating shape frequency or amplitude, there is a region where thresholds were unaffected (frequency: ≤RF4; amplitude: ≤0.05). Increasing either parameter beyond that critical value causes thresholds to rise. Thresholds increased with the square of shape frequency and approximately linearly with shape amplitude above the crucial value. Compared to the circle, thresholds are increased by about a factor of four for an RF13 and a factor of five for *A* = 0.3.

It is tempting to argue that the effect of RF on coherence thresholds may be due to sampling effects. The classical Nyquist limit requires at least two sampled elements per sinusoidal cycle of the RF pattern, resulting in a limit of RF3, 6, 9, 12, 15, and 18 for the 6 innermost rings with 6, 12, 18, 24, 30, and 36 elements. The Nyquist limit is typically derived for the values (positions) of a function. In our patterns, the position of elements was always on the sampled contour. Signal elements were defined by their orientations (being parallel to the contour). Hence, the signal elements carry two pieces of information: position and orientation (i.e., the functional value and its derivative at each sample point). This would double the Nyquist limit and guarantee sufficient number of elements for all tested radial frequencies with the single exception of RF ≥ 8 and the innermost ring. It is also clear that sampling limits did not affect the measurements for different shape amplitudes. The sampling limit does not change as a function of amplitude but the data show a strong dependence (Figure 3B). Moreover, the investigation of the performance for individual rings focused on rings 2 to 5, which are above the classical sampling limit for the shapes tested (RF0 and RF5). Considering these facts, it is seems unlikely that sampling limits have affected our data.

The dependence of coherence thresholds on shape frequency is consistent with the proposal that low RF patterns are processed more globally than high RF patterns. Psychophysical studies have repeatedly demonstrated that certain continuous and sampled RF patterns are globally processed (Wilkinson et al., 1998; Hess et al., 1999; Loffler et al., 2003; Bell et al., 2007; Dickinson et al., 2012; Schmidtmann et al., 2012). Global summation has been reported for low RF patterns (RF3, 5 and to some extent for RF10) but not for high RF patterns (e.g., RF24: Loffler et al., 2003; RF20: Schmidtmann et al., 2012). That detection thresholds in this study are similar for low RF patterns and increase for higher RF patterns supports the notion that signals from different parts of a contour are more efficiently pooled for low compared to high RF patterns. On average, twice as many signal elements are required to detect an RF10 pattern than e.g., an RF4 pattern.

Our data on shape detection like those obtained in studies on shape discrimination (typically with single closed contours) also show a dependence on shape amplitude. RF pattern discrimination thresholds have been shown to increase with increasing modulation amplitude (Bell et al., 2009; Schmidtmann et al., 2012). Thresholds for an RF5 pattern, with amplitudes in the range of those tested here, follow a power-law relationship with a slope of 0.55 (Bell et al., 2009). For shape detection, fitting a power law function to the data in Figure 3B (*A* ≥ 0.05) resulted in a slope of 0.77 ± 0.13 (CI), steeper than for RF discrimination.

### The role of local strategies: (1) contour strings

The dependence of detection thresholds on shape frequency and amplitude may be the result of a range of shape mechanisms that integrate information more or less efficiently along the circumference of the contour to which they are tuned. Other alternatives should, however, be considered. One feature that changes with increasing frequency and amplitude is the relationship between the orientations of adjacent elements along a contour. Given that the ability to detect a string of elements in noise has been shown to depend strongly on the relative orientation of neighboring elements (Field et al., 1993), it seems important to consider whether this feature can explain the current data. The orientation difference between adjacent elements on a circular contour depends on the number of elements that sample it. For example, in our experimental setup, a circle of radius 3.6° is sampled by 30 elements (ring 5) and each element differs in orientation from its two neighbors by 12°. If this orientation difference was responsible for shape detection, we should expect to see different coherence thresholds for circles sampled by different numbers of elements. This is clearly inconsistent with the data (Figure 4). Coherence thresholds for circles sampled by 12, 18, 24, and 30 elements (rings 2, 3, 4, and 5) yield similar thresholds despite their elements differing in orientation by 60, 20, 15, and 12°, respectively. Thus, a 2.5 fold change in inter-element orientation difference between the small and large circle is not reflected in the thresholds. This also holds for the RF5 data (Figure 4C).

A further argument against the role of inter-element orientation difference follows from the observed effect of increasing shape frequency and amplitude. Orientation difference increases by a factor of 1.7 when comparing a circle and an RF13 and by 1.9 when comparing a circle and an RF5 pattern with *A* = 0.3. The corresponding thresholds (Figures 3A,B) increased by factors of 4 and 5, respectively.

It has recently been proposed that RF discrimination may be linked to contour orientation. This suggestion is based on the observation that RF discrimination thresholds co-vary with the maximum orientation difference of an RF from circular (Dickinson et al., 2012). Dickinson et al. found that RF discrimination improves with shape frequency (cf. Wilkinson et al., 1998; Dickinson et al., 2012); in the present experiment, shape detection degraded with frequency. While there was no unmodulated circle for comparison in our experiments, we nevertheless examined whether local orientation difference between RF patterns and unmodulated circles could account qualitatively for our data.

The maximum orientation difference is a function of both shape amplitude (*A*) and frequency (ω), with the following relationship (Dickinson et al., 2012):

(4) $$\text{max}\left[\Delta_{\text{ori}}\left(\theta \right)=\arctan\left(\frac{r_{mean}\cdot A\cdot \omega \cdot \cos\left(\omega \cdot \theta \right)}{r\left(\theta \right)}\right)\right]$$

with θ being the angular component of the polar coordinate system and *r*_*mean*_ and *r* (θ) from Equation 1.

Fitting this function to the data for shape frequency or amplitude (Figure 3) provides a poor fit, arguing against the maximum orientation difference between an RF pattern and the corresponding circle as an explanation for the dependence of coherence thresholds on shape in our detection task.

It therefore seems unlikely that either the relationship between the orientations of adjacent elements or how much they differ from a circle are critical factors. Accordingly, we think it is doubtful that the rules derived from the psychophysical data on the grouping of adjacent elements into contour strings (Field et al., 1993) play a fundamental role in the detection of the sampled, closed contours in noise in our experiments. Any applicability of the association field model to our data is further complicated by the fact that the model requires neighboring elements to be linked into a contour. Interspersed noise elements with orientations differing from the contour path would dramatically reduce the visibility of the path. Our stimuli typically contained a number of randomly oriented noise elements between any two signal elements, an arrangement which disrupts the continuity of the contour. For the case of a circle, with average thresholds of between 4 and 5 elements for individual rings, this results in, for example, 5 signal elements per 24 elements on ring 4 at threshold. This is equivalent to 1 signal element per 4 noise elements or, on average, 4 noise elements separating two signal elements along the contour.

As pointed out by a reviewer, the possibility remains that some trials will contain adjacent signal elements along a contour ring. Observers could potentially use this as a cue. To directly investigate this possibility, we replicated experiment 2 but this time precluded the possibility of adjacent Gabors carrying signal. Consecutively numbered elements on each ring were divided nominally into odd and even numbered elements and signals randomly assigned to one of the groups. This guaranteed that two signal elements were separated by at least one noise element (up to the point of 50% coherence which was well above threshold for all tested conditions). All other experimental properties were identical to experiment 2. The results for two observers for circular and RF5 shapes and rings (2, 3, 4, 5) were compared to the data in the initial experiment. A repeated measures ANOVA with the two shapes (circle, RF5), various rings (2, 3, 4, 5) and the two configurations (with and without the possibility of adjacent signal elements) as factors revealed no significant differences between the two experimental configurations [*F*_(1, 3)_ = 4.388, *p* = 0.284]. Hence, imposing at least one noise element between any two adjacent signals does not negatively affect performance.

An additional possibility is that observers may use extended albeit interrupted contour segments. The main difference between this hypothesis and one that pools information across entire rings is the extent of global integration. There are two empirical arguments in favor of summation along the entire contour. Firstly, the number of signal elements at thresholds is small (e.g., 5 out of 30) and with it the probability of obtaining local signal strings. Secondly, the probability of obtaining local strings decreases with increasing number of elements on the ring but such a dependence of thresholds on ring radius is not reflected in the data. On balance, the evidence favors summation along entire contours.

Hence, despite a lack of contour strings, the contours are detectable, which can only be explained by highly global processes that have access to information from any part of the contour, and which do not rely on the relationship between adjacent element orientations.

### The role of local strategies: (2) local curvature

The relationship between adjacent elements also affects local curvature. Increasing the shape amplitude or frequency entails an increase in local curvature (convex and concave) and the change of curvature at points of inflections becomes more prominent. The important role of curvature extrema in object perception has been shown psychophysically (Attneave, 1954; Biederman, 1987) and been supported by physiological studies (Pasupathy and Connor, 2001). By showing that RF discrimination thresholds increase disproportionately when introducing small gaps at the peaks of RF patterns, Loffler et al. (2003) suggested that points of maximum convex curvature play a key role in RF discrimination. Findings from lateral masking experiments are consistent with the hypothesis of maximum curvature dominance (Habak et al., 2004) although other studies have highlighted a non-trivial role of other parts of the contour (Poirier and Wilson, 2007; Hancock and Peirce, 2008; Bell et al., 2010). If local curvature maxima were relied upon, one may expect shape detection sensitivity to increase with increasing shape amplitude and frequency as these manipulations increase the maximum curvature, but this is inconsistent with the data (Figures 3A,B). For detecting smooth contours in noise, a more appropriate assumption would be for local curvature to have the opposite effect, i.e., that coherence sensitivity is inversely proportional to the magnitude of curvature. The maximum curvature of an RF pattern is given by (Wilkinson et al., 1998; Bell et al., 2009):

(5) $$\kappa =\left(\frac{1+A+A\cdot \omega^{2}}{r_{mean}{\left(1+A\right)}^{2}}\right)$$

Fitting this equation to the data provides a good fit for shape frequency (*R*^2^ = 0.99; Figure 3A; gray line). It also predicts the observed dependence of coherence thresholds on amplitude (*R*^2^ = 0.98; Figure 3B; gray line) for amplitudes above the critical value of *A* = 0.05.

The existence of a range of shape frequencies and amplitudes, over which thresholds remain unaffected, can also be explained on the basis of local curvature. RF patterns with low amplitudes, such as those that are typically used for RF discrimination, contain only convex curvature. The amplitude at which an RF contour goes from only convex to contain a point of zero curvature is given by (Dickinson et al., 2012):

(6) $$A=\left(\frac{1}{1+\omega^{2}}\right)$$

Hence, where this transition occurs depends on both shape frequency and amplitude. Increasing amplitude and/or frequency beyond this point results in the contour containing concavities. For an amplitude of *A* = 0.05 (Figure 3A), the critical shape frequency for this transition is ω_crit_ = 4.36. For a frequency of RF5, the critical amplitude *A*_crit_ = 0.038. Both values are close to the data points where coherence thresholds relative to a circle start to increase (ω ≤ 4; *A* ≤ 0.05; Figure 3), suggesting that the transition from convex to concave may be an important factor in the processing of RF shapes (Kempgens et al., 2013).

It should be noted, however, that the design of our stimuli renders direct access to local curvature information largely unusable. This is because at least two adjacent signal elements are required for local curvature to be extracted from the display whereas signal elements will typically be separated by a number of noise elements. Hence, it is statistically unlikely that points of any particular curvature (e.g., maximum convex or concave, minimum) are covered by signal elements. Even when avoiding adjacent signal elements, performance is not negatively affected (see Control Condition above).

Overall our data show a dependence of coherence thresholds on the maximum curvature of the shapes to be detected. Increasing curvature by increasing shape frequency or amplitude inversely affects sensitivity. However, given the design of the stimuli, it is unlikely that the visual system is actually using local curvature directly. Rather, it suggests that the sensitivity of the global mechanisms underpinning shape detection in noise arrays has an inverse dependence on curvature.

### Texture vs. shape detector

To distinguish between two putative mechanisms that may underlie detection in this task, a texture detector or a shape detector (see Figure 1), we designed stimulus arrays in which signal elements were constrained to fall on one of a number of rings (Figure 4). For the example of a rotational texture mechanism, its threshold would be reached independent of the actual location of the signal elements within its receptive field as long as the orientations were concentric. The results of experiment 2, by contrast, show that if signal elements are constrained to specific annuli, the number of signal elements at threshold is lower than when they are spread across the display. This led us to hypothesize that multiple, concentric shape detectors, each tuned to a different diameter, underlies the high sensitivity seen for “textured” multi-element arrays such as the one used here and in previous studies (Braddick et al., 2000; Achtman et al., 2003).

This hypothesis can be tested by predicting performance when signal elements are spread randomly across the array based on the sensitivity when they are constrained to individual contours. If multiple, independent shape detectors, each limited by their own uncorrelated noise, were processing these arrays, the performance in the random positioned case should be predicted by the sensitivities of individual shape detectors. Assuming that their outputs are combined according to probability summation (Graham and Robson, 1987), this prediction is close to, but marginally underestimates performance (Figure 4) for circular as well as concentric RF5 shapes. As probability summation is a sub-ideal way of combining independent information, the results support a slightly more efficient strategy (Macmillan and Creelman, 1991; Nandy and Tjan, 2008; Machilsen and Wagemans, 2011; Gold et al., 2012). Irrespective of the details of the strategy, the data provide evidence for global shape mechanisms that integrate local information along contours rather than within a circular region.

It is possible that the different performance for the isolated and spread conditions may be in part due to lateral masking or attentional effects. Habak et al. (2004) measured RF pattern discrimination thresholds in the presence of larger masking patterns (lateral masking). Masking effects were strongest when the mask RF matched the test RF with regards to RF and phase (orientation). One might argue that such lateral masking effects may explain part of the decrease in performance when signal elements are distributed across four rings [2,3,4,5] compared to when they are restricted to individual rings (experiment 2). This is, however, unlikely for the circular contours. Circles have a limited masking effect on RF shapes (Habak et al., 2004) and it is unclear if lateral masking occurs for circular tests. Circles lack overall orientation and extreme points of curvature. Given that masking was shown to require curvature extrema of test and masking shapes to be aligned, one would expect little masking for circles. Based on the lack of lateral masking for circles and the similarity between the data for the circular and RF5 shapes (experiment 2), it seems unlikely that lateral masking has a significant effect on the conditions tested here. It remains therefore a possibility that lateral masking can impact negatively on the sensitivity of mechanisms that underlie contour discrimination but not contour detection.

Dickinson et al. (2009) have recently shown that selective attention can modulate sensitivity to circular Glass pattern detection: performance depends on observers' knowledge of the location of signal elements and is better when the location is known prior to the experiment. Our experiments were designed to avoid prior knowledge of signal location. Randomly interleaving conditions within a single block made it impossible for observers to know where signals would occur on individual trials. Consequently, observers were forced to attend to the entire stimulus array on every trial. This allowed direct comparisons to be made between performance when elements are located within individual rings and when elements are spread across rings, without the confound of an attentional advantage. It remains a possibility that the integration area for shapes embedded in noise is variable and under attentional control, as it is for Glass patterns (Dickinson et al., 2009).

A recent study, employing stimuli similar to ours, showed global signal integration for spiral textures. Webb et al. (2008) sampled spirals as well as concentric and radial forms. Global integration was evident for all tested forms. Backward masking had a selective effect on sensitivity and was strong when the form of the mask matched that of the target. This dependence was explained by a model consisting of multiple detectors, each broadly tuned to a specific spiral type. Apart from the concentric arrangement in Webb et al. (2008), which is similar to our circular condition with elements spread across rings, it is unclear if and how the other spiral configurations would tap into the mechanisms responding to the range of RF shapes tested here. Further studies are required to clarify if the same mechanisms are utilized for detecting spiral form and RF shapes. This said, the results from the earlier study confirms what our data here and others (Achtman et al., 2003) have shown: global pooling is effective for a range of sampled shapes and textures embedded in noisy arrays.

Overall, our data imply global shape detectors. The presence of a number of such mechanisms can explain the performance for a “textured” array. This is not to argue against the existence of texture mechanisms such as those proposed for Glass patterns (Wilson and Wilkinson, 1998). Rather, it suggests that texture mechanisms may have a lower sensitivity than shape detectors and that performance to “textured” Gabor arrays can, depending on the arrangement of the elements, be limited by multiple shape processes. The role of concentric shape detectors in the processing of Glass patterns remains a topic for future investigation.

### Tuning characteristics

Only about 5 signal elements were required to detect a contour embedded in ~150 noise elements. This was largely independent of the size of the contour and its shape (circle and RF5; Figure 4). This is a remarkably low number of elements and equates to a coherence level of 3%, substantially lower than what has been reported for detecting texture in noise (about 10%; Achtman et al., 2003), Glass patterns (15%; Wilson and Wilkinson, 1998; Dakin and Bex, 2002) or motion (10%; Newsome and Pare, 1988; Braddick et al., 2000). The fact that the absolute number and not the proportion of signal elements remains constant for contours of different sizes is also surprising. Intuitively, one would expect to find a constant signal-to-noise ratio of the elements within an annulus to which a particular detector is tuned. This would result in an increased number of signal elements with increasing radius as the number of total elements increases with size. Why the number of elements at thresholds is constant is unclear. One possibility is that sensitivity also depends on the average curvature of the shape, which is given by its radius, i.e., how flat or steep the contour is. If threshold signal-to-noise ratios were determined by the total number of elements on a contour as well as the average curvature, increasing the number of elements by increasing the size of the contour would be counter-balanced by lowering its curvature, resulting in a constant number of signal elements. This kind of effect, where two factors that each affect sensitivity cancel each other when co-varied, has been reported with circles (in this case number of elements and circle radius; Levi and Klein, 2000).

To further investigate the properties and tuning characteristics of putative shape mechanisms, orientation, and positional noise was added to the elements (experiment 3). Adding orientation jitter of ±13° had little effect on detectability suggesting that the underlying mechanism is insensitive to variations within this range. Further increasing orientation noise raised thresholds. These data are consistent with a mechanism that is broadly tuned to the orientation of local elements. The overall sensitivity of the mechanism is affected by the number of signal elements, as well as their orientations.

It is tempting to argue that the insensitivity to small changes in element orientation can explain why changes, within a certain range, in shape amplitude and frequency have no effect on performance (Figures 3A,B). Assume a single, general mechanism responsible for detecting a range of quasi-circular shapes (e.g., circle and RF5). Such a mechanism could be conceived as a global integrator that sums information from elements positioned broadly within an annular region and orientations that are approximately tangential to a circle. This process would respond equally well to circular contours and a range of RF shapes, as long as their frequencies and amplitudes were sufficiently low. A single mechanism responsible for the detection of a range of shapes is not, however, supported by the data. Consider an RF5 pattern with *A* = 0.05 (Figure 5). Depending on the precise location of an element, the orientations of the elements of an RF5 pattern differ from the orientations of corresponding elements on a circle. The orientations will differ most from a circle at the inflection points of the RF pattern, and be the same as that of a circle at corners and sides. For the amplitude tested in the experiment (*A* = 0.05), the orientations differ from those of a circle by up to ±14°. If a “circle” mechanism broadly tuned to local element orientation were confronted with an RF5 pattern, its sensitivity would be reduced in the same way as when adding orientation noise to the elements of a circle. Hence, one would expect the data for an RF5 pattern with *A* = 0.05 to be similar to those for a circle with added orientation noise of ±14°. Adding orientation noise to the RF5 pattern should further decrease sensitivity and the curve for orientation noise added to an RF5 pattern should be given by that for the circle shifted horizontally to the left by 14° in Figure 5. This is evidently not the case; instead the curves for the circle and the RF5 pattern are superimposed. Rather than a single mechanism responding to a range of shape, this argues in favor of separate shape mechanisms, each tuned to a particular shape with a similar, broad tuning profile for local orientation. The existence of multiple RF shape channels has support from studies on shape discrimination. Evidence from empirical results on subthreshold summation (Bell et al., 2008), adaptation (Anderson et al., 2007; Bell et al., 2008), and masking (Habak et al., 2004), as well as computational modeling (Poirier and Wilson, 2006), is converging in support of a number of narrowly tuned RF shape channels.

Applying positional jitter (experiment 3b; Figure 6) also affects detection. While low degrees of positional noise of up to 5% of the contour's radius left thresholds unaffected, largely eliminating any smooth contour path (radial variation of ±30%) lowered thresholds by about a factor of 3. The latter performance is similar to the condition where elements are randomly spread within the stimulus array (Figure 4 “all Rings”). This is consistent with the proposal that once the radial jitter exceeds the limit of the positional tuning of a mechanism, sensitivity will be given by the probability summation of multiple, independent detectors. The relatively stronger tuning for element orientation compared to position makes an interesting prediction. If elements were taken from an RF5 pattern and placed on the circumference of a circle, they should be perceived as an RF5 pattern rather than a circle. This prediction has recently been verified experimentally (Day and Loffler, 2009).

### Similarities and differences between RF discrimination and RF detection

There are a number of theoretical and experimental findings that question whether the data on detecting sampled RF shapes embedded in noise presented here are directly comparable to data on discriminating continuous RF shapes in the absence of external noise. For example, the detection of sampled RF shapes in noise shows a dependence on RF whereas RF discrimination does not (Wilkinson et al., 1998). Lateral masking affects RF discrimination (Habak et al., 2004) but no such effect is evident for RF detection (see above). One problem when comparing detection with discrimination data is related to the global pooling for RF shapes (Hess et al., 1999; Loffler et al., 2003; Bell and Badcock, 2008; Dickinson et al., 2012; Schmidtmann et al., 2012). For RF discrimination, the high sensitivity typically seen (in the hyperacuity range) requires information from different parts of the contour to be integrated. There has been a recent discussion as to whether sensitivity for RF discrimination improves gradually with the amount of contour information (e.g., Loffler et al., 2003; Dickinson et al., 2012) or requires information from all parts to be available simultaneously (Schmidtmann et al., 2012). Irrespective of this, it is clear that information from all parts of an RF contour is necessary to explain the exquisite sensitivity for RF discrimination. When measuring detection of sampled RF shapes in noise, this situation would only be available at a 100% coherence level. As detection thresholds are about an order of magnitude lower, observers are never presented with such a stimulus. Hence, if it was assumed that the mechanism governing RF discrimination was also responsible for RF detection, it would inevitably be stimulated sub-optimally within the measured range of coherence. The different sensitivities for RF detection and RF discrimination can be quantified by comparing our data from experiment 3 with those on RF discrimination. Adding orientation variance to the signal elements of up to ±13° did not adversely affect detection. When discriminating a continuous RF5 from a circle, the maximal difference in local contour orientation between the two shapes at threshold is less than 1° (assuming a threshold of *A* = 0.004; Dickinson et al., 2012). This equates to a 10-fold difference in orientation sensitivity between RF detection and discrimination. It is therefore unclear if the two tasks, detection and discrimination, are limited by different mechanisms or the same mechanism that operates in quite different regimes. It is, however, clear that both tasks are underpinned by global pooling of local information. In the case of RF discrimination, global pooling is required to account for the hyperacuity sensitivity. In the case of RF detection, global pooling is required to account for observer ability to detect patterns on the basis of a very small number of signal elements separated by multiple noise elements. Common to both, a range of mechanisms, tuned to different RF shapes, are required to explain behavioral data. Whether the same detectors are used for discriminating and detecting shapes or whether different or additional mechanisms are engaged, remains a question for future investigations.

In summary, for displays with sampled circular and non-circular contours, neither local inter-element interactions (association field), nor a texture mechanism (e.g., Glass pattern detector; Glass, 1969, Figure 1A), are sufficient to capture our data. Instead, our results are consistent with the existence of a range of highly sensitive, shape-specific analysers, which sum information globally but only within specific annuli (Figure 1B). These global mechanisms are tuned to the position and orientation of the local elements from which they pool information.

### Conflict of interest statement

The authors declare that the research was conducted in the absence of any commercial or financial relationships that could be construed as a potential conflict of interest.
